# From network pharmacology to molecular docking analysis of sterubin targets for Alzheimer

**DOI:** 10.6026/973206300200327

**Published:** 2024-04-30

**Authors:** Sittarthan Viswanathan, Thennavan Arumugam, Rengaraj Sivaraj, Srihari Subhashri Rajendran, Vimalavathini Ramesh, Kavimani Subramanian, A. Hannah Rachel Vasanthi

**Affiliations:** 1 Department of Pharmacology, Mother Theresa Post Graduate & Research Institute of Health Sciences (Government of Puducherry Institution), Puducherry - 605006, India; 2 Department of Pharmacology, Central Animal House, JIPMER, Puducherry - 605006; 3 Department of Pharmacology, Aarupadai Veedu Medical College & Hospital, Puducherry 607402; 4 Department of Biotechnology, Pondicherry University - 605014

**Keywords:** Network, pharmacology, molecular docking, sterubin, Alzheimer

## Abstract

Sterubin (7-O-Methyleriodicytol), a flavanone compound isolated from the leaves of Eriodicyton californicum and Eriodicyton angustifolium, has neuroprotective, anti-inflammatory, and antioxidant properties. Therefore, it is of interest to identify the potential targets for Alzheimer disease using network pharmacology. We report 25 overlapping targets among 100 potential targets of sterubin and 673 known targets of Alzheimer. APP, BACE-1, and AChE were among the ten hub targets enriched in biological processes and pathways relevant to Alzheimer's disease. Subsequent, molecular docking analysis shows that sterubin have optimal binding features with these hub gene targets for further consideration.

## Background:

Aging is commonly characterized by a gradual decline in various physiological functions, including cognitive ability, visual and auditory acuity, muscular strength, and sleep quality. This reduction is thought to result from loss of homeostatic balance within the body. Studies have suggested that aged brains experience various pathological changes including increased metabolic stress, reduced neurogenesis, and increased synaptic irregularities. Additionally, there is heightened expression of inflammatory markers and decreased expression of neuroprotective factors. Altered brain physiology, in conjunction with disruptions in the operation and synchronization of the circadian system, has been found to significantly enhance the occurrence of neurodegeneration, neurobehavioral insufficiencies, and cognitive aging [1].

Over the past 50 years, one of the primary objectives of pharmacological research on Alzheimer's disease (AD) has been the identification of cognitive enhancers [2]. AD is the 6th leading cause of death in the USA in 2019, In the years 2020 and 2021, COVID-19 attained a position among the top ten causes of mortality, and it ranked as the seventh-highest cause of death, costing over $232 billion annually, making it burdensome after cancer and heart disease [3]. As of 2021, approximately 5.8 million individuals aged 65 years and above in the United States are living with AD. The disease is the primary cause of dementia in the aging population, affecting over 55 million people worldwide. According to the 2019 World Alzheimer's Report, it is projected to increase to 88 million by 2050 [4]. AD is the most prevalent type of dementia and is characterized by persistent deterioration in cognitive abilities, behavior, social skills, and capacity to carry out daily activities independently. The etiology of AD is attributed to the accumulation of amyloid β and phosphorylated τ protein aggregates in the brain, resulting in neuronal degeneration of neurons [5]. Several hypotheses have been proposed to explain the cause of AD. Currently approved drugs for AD include cholinesterase inhibitors (donepezil, rivastigmine and galantamine) and NMDA receptor antagonist (memantine) [6]. Only two drugs (Aducanumab and Lecanemab) have been approved by the FDA for the past 21 years [7, 8]. Each year, numerous drugs have been developed to treat AD in the hope of achieving successful outcomes; however, the majority of these attempts were unsuccessful at the preclinical stage, prior to the initiation of clinical trials [9].

Historically, various plant sources have been utilized to address learning and memory impairments. Additionally, there has been increasing interest in the potential benefits of natural resources in treating cognitive impairments, including AD, along with their associated pathogenesis [10]. Initially, alkaloid-containing plants were the primary focus of this study; it is well established that alkaloids strongly interact with receptors in the central nervous system. However, in recent years, there has been a shift towards studying flavonoids, which have been shown to be effective in preventing symptoms associated with neurodegenerative diseases such as Alzheimer's disease and Parkinson's disease. Flavonols, flavanones, flavanones, anthocyanins, isoflavones, and flavan-3-ols are the main flavonoids that possess neuroprotective properties [11].

Sterubin (7-O-Methyleriodicytol) is a flavanone compound that was first isolated from the leaves of Eriodicyton californium, Eriodicyton angustifolim (Yerba santa). It has a broad range of pharmacological properties such as high neuroprotective, anti-inflammatory, anti-oxidant, and anti-amyloid properties, and is used to treat respiratory ailments such as cough, cold, asthma, bronchitis and age-related complications. Sterubin has been identified through old-age-associated phenotypic screening [12]. The immense pharmacological properties of sterubin make it a valuable and interesting compound. However, the molecular mechanisms responsible for this biological potential have not yet been systematically evaluated. Sterubin, which is a potent antioxidant, anti-cholinesterase, anti-aging, neuroprotective, anti-inflammatory and neurotrophic roles, ameliorating learning and memory, anti-amyloidogenic effects, suppressing the activation of microglia, and mediating inflammatory processes in the central nervous system (CNS) [13].

Network pharmacology is a new in silico drug discovery approach developed by Hopkins in 2007 to identify active compounds and putative molecular targets in a broad range of herbal formulae or simple herbs [14]. This tool operates based on systems biology and integrates multiple approaches, including poly-pharmacology, molecular network analysis, bioinformatics, and computer simulations. This strategy not only accelerates drug discovery but also saves time, energy, and money [15]. Network pharmacology involves the identification of genes related to compounds and diseases, the construction of a protein-protein interaction (PPI) network, and ultimately, the analysis and visualization of the network. The process begins with the construction of molecular networks from large databases, followed by identification of key nodes and biological pathways using network analysis. Finally, the network undergoes additional validation to confirm the interactions between the most active components and their potential targets [14]. Therefore, it is of interest to use network pharmacology (Figure 1 - see PDF) to investigate the mechanisms underlying the therapeutic effects of sterubin in AD's.

## Methodology:

## Pharmacokinetics properties and toxicity prediction:

The PubChem database, which can be accessed at https://pubchem.ncbi.nlm.nih.gov [16], was used to retrieve the canonical SMILES of sterubin. SwissADME, a tool available at http://www.swissadme.ch [17], was used to analyze the drug likeness and physicochemical properties of sterubin, including its ADME properties. Finally, the toxicity of sterubin was assessed using OSIRIS, a tool available at https://www.cheminfo.org/flavor/cheminformatics/Utility/Property_explorer/index.html [18].

## Swiss Target Prediction:

Swiss target prediction http://www.swisstargetprediction.ch [19], an online platform designed for predicting the targets of small bioactive molecules, was employed to identify potential targets for sterubin. By utilizing this tool, the SMILES data of sterubin were imported into Swiss Target Prediction, with the species set to Homo sapiens. Predictions of potential targets were collected and analyzed. Swiss-target prediction is widely recognized as the leading software for determining the most likely protein targets of bioactive chemicals.

## Disease-Target Prediction:

The potential targets were selected from GeneCards (http://www.genecards.org) [20] and DisGeNET (http://www.disgenet.org) [21] using the keyword "Alzheimer's disease". The target's standard name was obtained from UniProtKB, specifying the organism as "Homo sapiens." The DisGeNET database was utilized to determine the gene-disease association (GDA) score, which was used to rank the association between genes and AD. In this study, targets with a GDA score greater than 0.1 were considered to be highly correlated with AD. The relevance score threshold for the targets in the GeneCards database was set to a minimum of 20. The two databases were subsequently combined, taking into account their respective targets. Furthermore, any duplicate genes were removed from the analysis [22].

## Intersection of related targets:

To more accurately assess the connection between AD-related targets and sterubin targets, we merged the two sets of targets and created Venn diagrams using an online tool from http://bioinformatics.psb.ugent.be/webtools/Venn [23]. The overlapping targets were selected for further analysis as potential therapeutic targets.

## Construction and analysis of PPI network:

The overlapping targets were then imported into STRING database version 11.0 to construct a PPI network https://string-db.org/[24]. The criteria for selecting the human organism were a minimum interaction score of greater than 0.4. Only interactions that met this criterion were deemed significant. Protein-protein interaction (PPI) networks are composed of nodes that signify target proteins and edges, which symbolize the interactions between proteins. The thickness of an edge is proportional to the combined score of the interaction. The degree of a node refers to the number of other nodes directly connected to it. A higher degree indicated a more important node. Following its development, this network was subsequently imported into Cytoscape (Version 3.7.2) for visualize and analyze its structure. The Cytoscape software may be obtained by visiting the Cytoscape website https://cytoscape.org/ [25]. The degree was calculated to identify core targets using CytoHubba. In this study, the top ten proteins ranked by degree were selected and designated as core targets [26].

## GO and Kyoto Encyclopedia of genes and genomes (KEGG) enrichment analysis:

The analysis of gene ontology and KEGG enrichment pathways was conducted utilizing the Database for annotation, visualization, and integrated discovery (DAVID), which is available at https://david.ncifcrf.gov/ [27]. The DAVID functional annotation tool was utilized to allocate functional roles at three levels - cellular component (CC), molecular function (MF), and biological process (BP) - to a selection of critical genes. DAVID is a functional enrichment database accessible through the Web, enabling researchers to comprehend the bioactivity of a multitude of genes. In the current study, a significance level of ≤ 0.05 was established, and the top ten GO enrichments and top ten KEGG pathways were selected for further analysis. These results were then visualized using an online tool available at http://www.bioinformatics.com.cn [28].

## Molecular docking:

Molecular docking is commonly used to validate the interactions between target proteins and ligands. In this case, the ligand (sterubin) was docked with the top ten potential targets. The structures of sterubin were retrieved from the PubChem database. The selected 3D structure of the ligands was retrieved from the PubChem compound database in SDF format, followed by conversion to PDB format and optimization using Bio-Discovery Studio. Protein Data Bank https://www.rcsb.org/ [29] was used to obtain the crystal structures of top ten target genes. Prior to docking analysis, prominent active site prediction of top ten selected targets was carried out by PDB Sum database https://www.ebi.ac.uk/thornton-srv/databases/pdbsum/ [30]. Molecular docking was carried out using Auto dock 4.2.1 software based on Lamarckian Genetic Algorithm was used to determine the appropriate binding modes of ligands. Grid maps were generated by Auto Grid program. Each grid was cantered at the crystal structure of the corresponding targets. A grid box with a dimension of 60 Å X 60 Å X 60 Å and spacing of 0.375 Å. For all ligands, random starting positions, random orientations, and torsions were used. The Docking parameters Number of Genetic Algorithm (GA) runs: 25, Population size: 150, Maximum number of evaluations: 2,500,000, Maximum number of generations: 27,000 were used for this study. All the others parameters were set as defaults. The structure with the lowest binding free energy and the most cluster members was chosen for the optimum docking conformation [31, 32].

## Results:

## Pharmacokinetic properties and toxicity prediction of sterubin

The structural information of sterubin was obtained from PubChem shown in Figure (see PDF), and the relevant ADME information was obtained from SwissADME. Table 1 displays the SwissADME predicted pharmacokinetic of sterubin. Sterubin complies with Lipinski rule of 5 and is predicted to have a good drug-likeness. The OSIRIS software was employed to evaluate the toxicological profile of sterubin, and the results indicated that sterubin does not possess tumorigenicity, mutagenicity, irritant or reproductive toxicity. Consequently, the findings suggest that sterubin is devoid of observable toxicity.

## Potential Targets:

We obtained a total of 100 sterubin target genes from Swiss Target Prediction and 648 AD-related targets from DisGeNET and GeneCards. Based on the above results, we identified 25 targets of sterubin against AD by overlapping of 100 sterubin associated targets and 648 AD related targets shown in Figure 3 (see PDF)

## Construction and analysis of PPI network of sterubin:

25 overlapped targets were uploaded to STRING database to identify the interactions. Then, we constructed a PPI network (Figure 4 - see PDF) consisting of 25 nodes and 79 edges, average node degree 6.32. After visualizing the PPI network in Cytoscape, CytoHubba plugin was utilized to find the Hub genes. The plugin offers twelve topological methods of analysis, from which the degree method was selected to predict Hub genes. The degree method is based on the highest degree of connectivity between targets, indicating that genes with the highest degree are likely to be key targets due to their increased connectivity with other genes. Top ten targets (APP, BuChE, TNF-α, AChE, GSK-3β, ESR-1, PPARG, BACE-1, MMP9 and MOA-B) are shown in Figure 5(see PDF).

## KEGG pathway and GO analysis:

We utilized the DAVID database to analyze the potential 25 target genes for enrichment in GO and KEGG pathways. According to GO function analysis the top ten target of BP, MF and CC categories were chosen based on P<0.05, as shown in Figure 6 (see PDF). The Benjamini-Hochberg process was employed to correct the p-values for BP (90), CC (26) and MF (25), respectively. Target protein in the BP category were mainly involved cellular response to beta-amyloid, response to xenobiotic stimulus, negative regulation of pri-miRNA transcription from RNA polymerase II promoter, regulation of catalytic activity, cognition, positive regulation of protein phosphorylation, acetylcholine catabolic process and synapse organization. MF few examples are enzyme binding, identical protein binding, beta-amyloid binding, peptidase activity, estrogen receptor binding, protein homodimerization activity, RNA polymerase II transcription factor activity, acetylcholinesterase activity, collagen binding and cholinesterase activity. Then finally CC such as cell surface, extracellular exosome, platelet alpha granule lumen, synapse, peptidase inhibitor complex, membrane, extracellular space, plasma membrane and extracellular region.

According to KEGG pathway analysis predicted 14 pathways regarding the Anti-Alzheimer targets. Ten KEGG pathways were associated with the targets genes (p<0.05) shown in bubble plots of bioprocess and pathways were drawn by uploading the data to the bioinformatics platform (Figure 7 - see PDF) as well as their enrichment ratios. Alzheimer's disease (hsa05010), estrogen signalling pathway (hsa04915), pathways in cancer (hsa05200), arachidonic acid metabolism (hsa00590), chemical carcinogenesis-receptor activation (hsa05207), lipid and atherosclerosis (hsa05417), AGE-RAGE signaling pathway in diabetic complications (hsa05417), pathways of neurodegeneration-multiple disease (hsa05022), cholinergic synapse (hsa04725) and serotonergic synapse (hsa04726) these pathways were significantly enriched.

## Compound-pathways-targets network:

We created a Drug-Pathway-Target network diagram to more clearly show how sterubin, targets, and pathway interact depict in Figure 8 (see PDF) using Cytoscape 3.7.2. Fourteen pathways, 25 core common targets with sterubin were connected. The network contained 41 nodes and 90 edges, in which the yellow shape represented the compound, targets were represented in green square and pathways using rose rectangular shape.

## Molecular docking analysis:

For the molecular docking, ten target genes (APP, BuChE. TNF-α, AChE, GSK-3β, ESR-1, PPARG, BACE-1, MMP9 and MOAB) were selected by comparing the hub genes with results provided by KEGG analysis in the pathway of Alzheimer's disease. As shown in Table 2, among the ten targets, (APP, BACE-1, AChE, BuChE, and TNF-α) showed the best interaction and lowest binding affinities towards sterubin compared to donepezil. According to receptor-ligand docking theory, it is generally accepted that the docking energy is inversely proportional to the binding affinity. Specifically, a more negative docking energy suggests a stronger binding affinity between the protein and the ligand [33].

## Discussion:

The treatment of AD presents a significant challenge due to its complex pathology. Single-target drugs or those that focus on a single pathway may not be sufficient to achieve the desired therapeutic effects. Investigating AD pathology and developing novel anti-Alzheimer drugs can be facilitated through the utilization of network pharmacology approaches in conjunction with various natural products. These approaches hold great potential for addressing the complex nature of AD and may lead to more effective treatments [34]. Network pharmacology combines computational, experimental, and clinical approaches to study the pharmacological mechanisms of natural products. This integrative approach creates optimal conditions for exploring the complex interactions of natural products with biological systems. This shift has moved us from a "one-target, one-drug" approach to a "multiple-target, multiple-component-therapeutics" approach [35]. Sterubin is derived from the leaves of Eriodicyton californium and Eriodicyton angustifolium. Previous studies reported that it have a significant antioxidant activity, protecting against oxytosis in HT22 cells and energy loss in PC12 cells. It also exhibits potent anti-amyloid activity. It protects against multiple inducers of cell death, activating distinct death pathways. Sterubin strongly induces the antioxidant transcription factor Nrf2 and exhibits robust anti-inflammatory activity. Additionally, it has anti-hair greying properties and can prevent Aβ-induced decreases in short and long-term memory in a short-term model of AD [36].

In the present study, we determine the pharmacokinetic properties, toxicity prediction, potential targets, and PPI network analysis of sterubin in relation to Alzheimer's disease (AD). Sterubin complies with Lipinski's rule of 5 and is predicted to have good drug-likeness and also free form toxicity. The study identified 25 targets of sterubin against AD by overlapping 100 sterubin-associated targets and 648 AD-related targets. A PPI network consisting of 25 nodes and 79 edges was constructed, and the hub genes were identified using the CytoHubba plugin. The top ten targets were screened according to the degree and they are APP, BuChE, TNF-α, AChE, GSK-3β, ESR-1, PPARG, BACE-1, MMP9 and MOAB.

According to GO and KEGG pathway analyses revealed that the top ten targets were involved in various biological processes, molecular functions, and cellular components. KEGG pathway analysis predicted 14 pathways related to Anti-Alzheimer targets, with ten pathways significantly enriched. A drug-target-pathway network diagram was created using Cytoscape to show the interactions among sterubin, targets, and pathways. By molecular docking analysis revealed that five targets (APP, BACE-1, AChE, BuChE, and TNF-α) showed good binding affinity towards sterubin compared to donepezil. The range of binding score of APP -9.78 Kcal/mol, BACE-1 -8.89 kcal/mol, AChE -11.03 kcal/mol, BuChE -8.37 kcal/mol and TNF-a -8.90 kcal/mol. The present research provides a comprehensive overview of sterubin, detailing their potential targets and the pathways involved in treating AD through network pharmacology. This serves as a foundation for future experimental research.

## Conclusion:

We utilized network pharmacology and database mining to detect molecular targets (APP, BACE-1, AChE, BuChE, and TNF-α) for sterubin for Alzheimer's disease. Molecular docking analysis data shows that sterubin have optimal binding features with these hub gene targets for further consideration.

## Disclosure statement:

The authors declare no conflicts of interest.

## Ethical approval:

This article does not contain any human participant and animal work.

## Author contributions:

All the authors contributed equally to this work.

## Funding:

Self-funding.

## Data availability statement:

All dataset supporting this article is available within the article and its supplementary files.

## Figures and Tables

**Table 1:** Molecular properties of sterubin

**Properties**	**Sterubin**
Molecular formula	C16H14O6
Molecular Weight	302.28
Hydrogen Bond Donor	3
Hydrogen Bond Acceptor	6
Rotatable bond	2
Topological Polar Surface Area (Å)	96.22
Drug likeness	Good
Lipinski	Yes
GI absorption	High
Clog P	2.09
Solubility log	-2.66
BBB	No
Log Kp (skin permeation)	-6.48 cm/s

**Table 2:** Binding energy of sterubin and potential targets protein

**Target**	**PDB ID**	**Binding Energy Affinity (Kcal/mol)**	
		**Sterubin**	**Donepezil**
APP	3PMR	-9.78	-8.33
BACE-1	5HDZ	-8.89	-7.47
AChE	4EY7	-11.03	-8.84
BuChE	4B0P	-8.37	-7.9
TNF-α	2FV5	-8.9	-7.5
GSK-3β	1Q5K	-7.5	-8.85
ESR1	3ERT	-6.34	-7.34
PPARG	8B8W	-7.95	-7.03
MAOB	2Z5Y	-8.87	-7.56
MMP9	5TH6	-9.34	-6.32
